# The Effects of Different Types of Dual Tasking on Balance in Healthy Older Adults

**DOI:** 10.3390/jpm11090933

**Published:** 2021-09-18

**Authors:** Graça Monteiro de Barros, Filipe Melo, Josefa Domingos, Raul Oliveira, Luís Silva, Júlio Belo Fernandes, Catarina Godinho

**Affiliations:** 1 Fisio-Lógica Centro de Fisioterapia, Lda, 1350-275 Lisboa, Portugal; gracamantua@gmail.com; 2 Escola Superior de Saúde Atlântica, 2730-036 Barcarena, Portugal; 3 Laboratório de Comportamento Motor, Faculdade de Motricidade Humana, Universidade de Lisboa, 1495-688 Cruz Quebrada, Portugal; fmelo@fmh.ulisboa.pt; 4 Grupo de Patologia Médica, Nutrição e Exercício Clínico (PaMNEC) do Centro de Investigação Interdisciplinar Egas Moniz (CiiEM), Escola Superior de Saúde Egas Moniz, Caparica, 2829-511 Almada, Portugal; domingosjosefa@gmail.com (J.D.); juliobelo01@gmail.com (J.B.F.); 5 Neuromuscular Research Lab, CIPER, Faculdade de Motricidade Humana, Universidade de Lisboa, 1495-688 Cruz Quebrada, Portugal; roliveira@fmh.ulisboa.pt; 6 Physics Department, LIBPhys-UNL, Nova School of Science and Technology, Universidade Nova de Lisboa, Caparica, 2829-516 Almada, Portugal; lmd.silva@fct.unl.pt

**Keywords:** dual-tasking, cognitive function, postural control, older adults

## Abstract

Numerous of our daily activities are performed within multitask or dual task conditions. These conditions involve the interaction of perceptual and motor processes involved in postural control. Age-related changes may negatively impact cognition and balance control. Studies identifying changes related to dual-task actions in older people are need. This study aimed to determine the effects of different types of dual-tasking on the balance control of healthy older adults. The sample included 36 community-living older adults, performing two tests—a sway test and a timed up-and-go test—in three conditions: (a) single motor task; (b) dual motor task; and (c) dual motor task with cognitive demands. Cognitive processes (dual-task and cognition) affected static balance, increasing amplitude (*p* < 0.001) and frequency (*p* < 0.001) of the center of mass displacements. Dynamic balance revealed significant differences between the single motor condition and the other two conditions during gait phases (*p* < 0.001). The effect of dual-tasking in older adults suggests that cognitive processes are a main cause of increased variability in balance and gait when under an automatic control. During sit-to-stand, turning, and turn-to-sit movements under dual-tasking, the perceptive information becomes the most important focus of attention, while any cognitive task becomes secondary.

## 1. Introduction

Daily life is occupied by dual-tasking behaviors, such as walking while talking with someone or while taking a picture on the phone. Effective daily functioning requires people to share their attention resources between the cognitive and the postural requirements necessary to complete the tasks [1]. This ability to perform concurrent performances is known as dual-tasking. A decreased capacity to perform dual-tasking may reduce the person’s ability to participate in their life roles [2]. Due to the aging process and prevalence of chronic diseases, older adults show some levels of decline when performing postural tasks while dual-tasking [3]. Upholding and improving older adults’ ability to perform under dual-task situations is an imperative goal for extending their functionality.

The literature reports several factors that are considered to influence the person’s ability to divide their attention resources between the two tasks, namely external factors, such as the tasks’ complexity, and intrinsic factors, such as physical status, executive function, and task prioritization [4,5].

Activities such as driving or walking are considered automatic motor sequences, largely operating independently from more cognitively intensive processes such as communication. When combined into a dual-task activity, the interaction of the perceptual-motor and cognitive neurophysiologic processes may have an influence upon the postural control that has a primary support function [6,7]. The turning characteristics during ambulating are a major contributor to motor disability, falls, and reduced quality of life in older people, largely because the necessary (intrinsic) dynamic balance control decreases with age [8].

Previous research indicates that postural control and gait in older adults lose some level of automaticity, defined as the capacity to be independently executed with minimum attentional costs, and indicated by alterations of motor patterns during dual-task activities [9,10]. The degree to which postural control and gait change during the simultaneous performance of other tasks is considered related to the level of difficulty of the concurrent task [3]. 

Questions remain regarding the overall amount of influence of different dual-task combinations (motor–motor versus motor–cognition) on postural control and gait. Although different aspects of the influence that dual-task activities have on postural control and gait have been observed in healthy older adults [11,12], the factors that contribute to postural control and gait changes, in response to “dual-tasking” among this population have not been fully clarified. It is hypothesized that in a homogeneous sample of healthy older adults with no perturbation in mobility and cognitive function, postural control and gait will remain under greater automatic control and thus, dual-task decrement, although present, will remain at a reduced level.

It is of great relevance to develop a normative evaluation of the postural control during static and dynamic equilibrium conditions [13]. This evaluation should include the performance of single and dual-tasks in order to determine which parameters may be altered with aging.

Postural and gait analysis obtained during the execution of different motor tasks will allow for the understanding of the interaction between motor and cognitive capacity, as well as the effects upon healthy aging subjects [14].

The objective of this study was to evaluate the effects of dual-task activities on balance control in healthy older individuals, namely, by exploring its influence on the temporal and space parameters of static activities such as postural sway in addition to dynamic tasks (standing and sitting on a chair and walking in conjunction to rotational movements) while performing a balance control task such as carrying a tray with a full glass of water. 

## 2. Materials and Methods

### 2.1. Study Design

This investigation was based on an observational cross-sectional study with a single moment evaluation. 

### 2.2. Sampling and Recruitment

Healthy older adults were recruited from the community by advertisements on social media and at seniors’ centers by phone or in person. The sampling method selection was non-probabilistic by convenience.

Subjects were included if they were seniors—aged 65 years and over, independent in their daily activities, and presenting independent walking.

Subjects were excluded if they presented vestibular disorders, neurological diseases, lack of cognitive skills according to the results of Mini Mental Score (MMS) < 20, musculoskeletal impairments that could affect gait, and inability to stand and walk unassisted. 

### 2.3. Ethics and Procedures 

This study follows the principles of the Declaration of Helsinki. All participants provided written informed consent and the study was approved by the Ethics Council of the Faculty of Human Kinetics (ID: CEFMH N°4/2016 in 15 February 2016).

### 2.4. Data Collection

Subjects were asked to perform two different tests randomly—the instrumented sway test (ISWAY) and the instrumented timed up-and-go test (ITUG)—during three task conditions that were applied randomly: (a) single motor task; (b) dual motor task (single motor task carrying a tray with a full glass of water); and (c) dual motor task with cognitive demands (the same as the dual motor task including counting back from 100, three by three). During the ISWAY test, subjects had to maintain a stable standing position.

**Figure 1:**
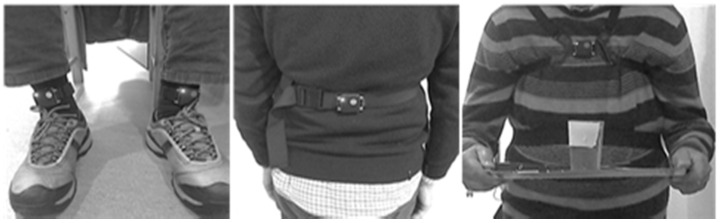
Placement of the Opal sensors and tray with a glass of water.

Balance and gait were measured using four Opal inertial sensors and automated algorithms from Mobility Lab, by APDM (APDM Inc., Portland, OR, USA). Sensors were placed on both ankles, the chest, and the posterior trunk at the level of L5 (Center of Mass—CM), with elastic Velcro bands sufficiently stable to avoid any undesirable dress movement (Figure 1). 

Inertial sensor data was collected and wirelessly streamed to a laptop for automatic generation of gait and balance metrics provided in specific reports by the Mobility Lab software. In the ISWAY test, subjects were asked to stand quietly for 30 s. In the ITUG test, subjects had to stand up from a chair without using their arms, walk 7 m, turn around to walk back to the chair, and sit down. Subjects repeated the ITUG test three times, once for each test condition (single motor task, dual motor task and, dual motor task with cognitive demands). Before the tests, each subject was submitted to a familiarization trial.

### 2.5. Data Analysis

The APDM automated analysis algorithm identified the standing position of the ISWAY test as well as the sit-to-stand, gait, turning and turn-to-sit components/phases of the ITUG test, providing a total of 52 spatial-temporal metrics [15]. From this automatic analysis, we monitored the following measures: Postural Sway (ISWAY test)—Forward/Backward and Right/Left amplitude (cm), Frequency (Hz), and Ellipse Sway area (m^2^/s^4^); ITUG: Sit-to-Stand—Time duration (s), Peak velocity (^0^/s), and Trunk range of movement (RoM) (^0^); Gait—Duration (s), Stride length (% of height), Cadence (steps/min), Stride velocity (% of height/s), Double support time (% of total gait cycle duration), Swing phase (% of total gait cycle duration), and Stance phase (% of total gait cycle duration); Turning—Duration (s), Number of steps (n°), Turn Peak velocity (^0^/s), and Step duration during turning (s); Turn-to-Sit—Duration (s), Turn Peak velocity (^0^/s), and Trunk range of movement (RoM) (^0^). 

Data results were subjected to analysis to identify the presence of any outliers. The assumption of normality was verified by Kolmogorov–Smirnov test. Kurtosis and skewness of the distributions were also analyzed. A repeated measures ANOVA was used for comparisons among the three conditions—(1) single motor task, (2) dual motor task, and (3) dual motor task with cognitive demands. 

Sphericity was verified by Mauchly’s test, and when this assumption could not be accepted, this parameter was corrected through the Greenhouse–Geisser Epsilon. Significant differences in ANOVA led to multiple comparisons with Bonferroni adjustment. Friedman’s test was applied when the normality assumption was not verified, and in case of significant differences, Dunn’s multiple comparisons were applied. The level of significance was set at 0.05. Data analysis included kinematic parameters during the two different tests (ISWAY and ITUG) during the three different conditions (single motor task, dual motor task, and dual motor task with cognitive demands). 

## 3. Results

### 3.1. ISWAY Test

**Figure 2:**
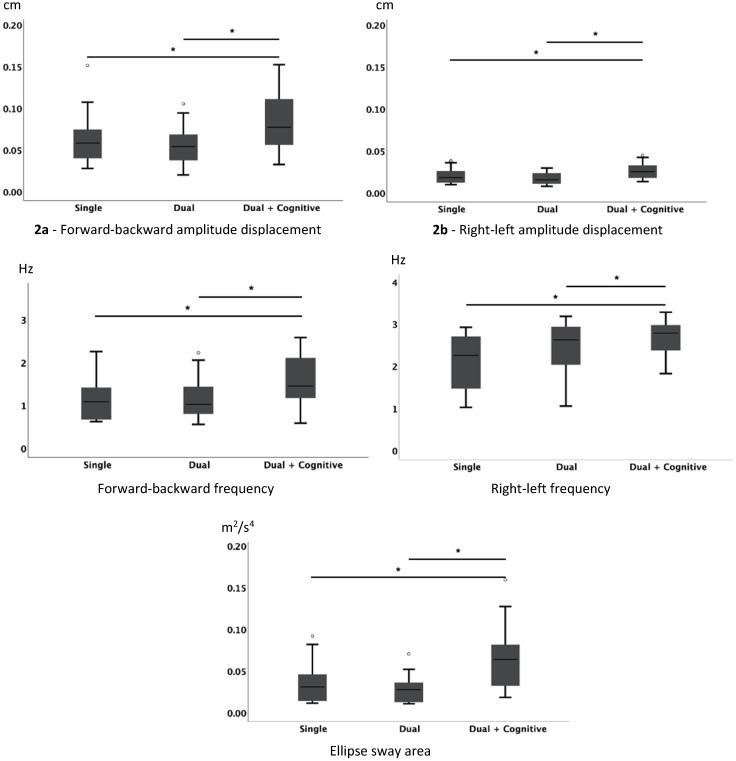
Balance parameters for the three conditions: single task, dual task, and dual and cognitive task during the ISWAY test. * Significant differences for pairwise comparisons.

Thirty-six healthy older adults (9 men and 27 women, 73 ± 5.7 years) were recruited from the community. All participants consistently altered their static balance and gait pattern in response to additional dual-task load, although specific kinematic changes varied according to task conditions. Postural control in standing position, measured via the ISWAY, was only affected during the dual motor task with cognitive demands, due to the increased cognitive complexity when compared with the other two conditions (single task and dual motor task). There were no differences in kinematic data between the other two motor tasks (single and dual motor tasks). Specifically, instrumental assessments related to the ISWAY test showed an increase in the postural sway parameters of the center of mass (CM) movement in terms of forward–backward (*χ*^2^ (2) = 19.385; *p* < 0.001; *N* = 26), and right–left (𝐹_(2,50)_ = 18.956; *p* < 0.001) displacement amplitude, forward–backward (*χ*^2^ (2) = 20.846; *p* < 0.001; *N* = 26) and right–left (*χ*^2^ (2) = 22.020; *p* < 0.001; *N* = 26) displacement frequency, and ellipse sway area (𝐹_(1.488, 37.194)_ = 27.361; *p* < 0.001). Figure 2 shows significant differences in pairwise comparisons for ISWAY test parameters and the respective boxplots.

### 3.2. ITUG Test

The dynamic balance analyzed during the ITUG test revealed highly significant differences (*p* < 0.001) between the single motor condition and the other two dual task conditions (dual motor task and dual motor task with cognitive demands) in the kinematic data of the ITUG test different phases.

#### 3.2.1. Sit-to-Stand Phase

With respect to time duration, there were no significant differences among the different test conditions, but there were highly significant (*p* < 0.001) differences in peak velocity (*χ*^2^ (2) = 46.545; *N* = 33) and trunk range of movement (RoM) (*F*_(2,64)_ = 41,861; *p* < 0.001) between the single motor task and the other two tasks (dual motor task and dual motor task with cognitive demands) although there were no statistical significant differences between the two dual-task activities.

**Figure 3:**
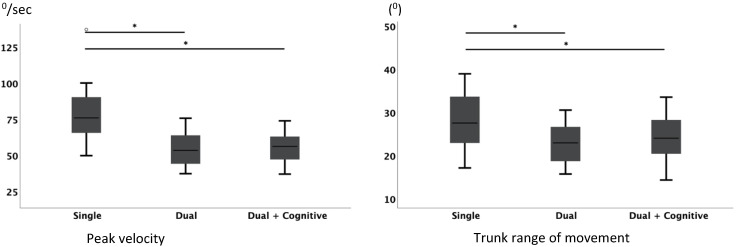
Sit-to-stand parameters for the three conditions: single task, dual task, and dual and cognitive task during the ITUG test. * Significant differences for pairwise comparisons.

Figure 3 shows significant differences in pairwise comparisons for sit-to-stand parameters and the respective boxplots.

#### 3.2.2. Gait Phase

Significant differences in time duration were observed among all three conditions (*F*_(1.681, 58.831)_ = 61,377; *p* < 0.001). Since there were significant differences between all conditions, showing that the gait duration increases as the level of complexity of the task also increases.

Stride length revealed significant differences among all conditions (*F*_(2, 70)_ = 39.375; *p* < 0.001), again with the reduction in stride length relative to the level of complexity of the task.

The cadence parameter demonstrated significant differences between the dual motor task with cognitive demands condition and the other two conditions (single motor task and dual motor task) (*F*_(1.408, 49.281)_ = 41.767; <0.001). There were no significant differences between the other two conditions.

Stride velocity showed significant differences among all task conditions (*F*_(1.590, 55.665)_ = 54.459; *p* < 0.001), with a reduction of the results related to the level of complexity of the task.

Double support time revealed significant differences between the single motor task and the dual motor task with cognitive demands condition (*χ*^2^ (2) = 44.169; *p* < 0.001; *N* = 36). No significant differences were observed between the other two conditions.

Similarly, assessment of swing phase only demonstrated significant differences between the single motor task and the dual motor task with cognitive demands condition (*F*_(1.672, 58.529)_ = 48.018; *p* < 0.001), showing a marked reduction in swing time. No significant differences were found between the other two conditions.

Stance phase showed significant differences between the single motor task and the dual motor task compared to the dual motor task with cognitive demands condition (*χ*^2^ (2) = 43.504; *p* < 0.001; *N* = 36). No significant differences were observed between the other two conditions.

**Figure 4:**
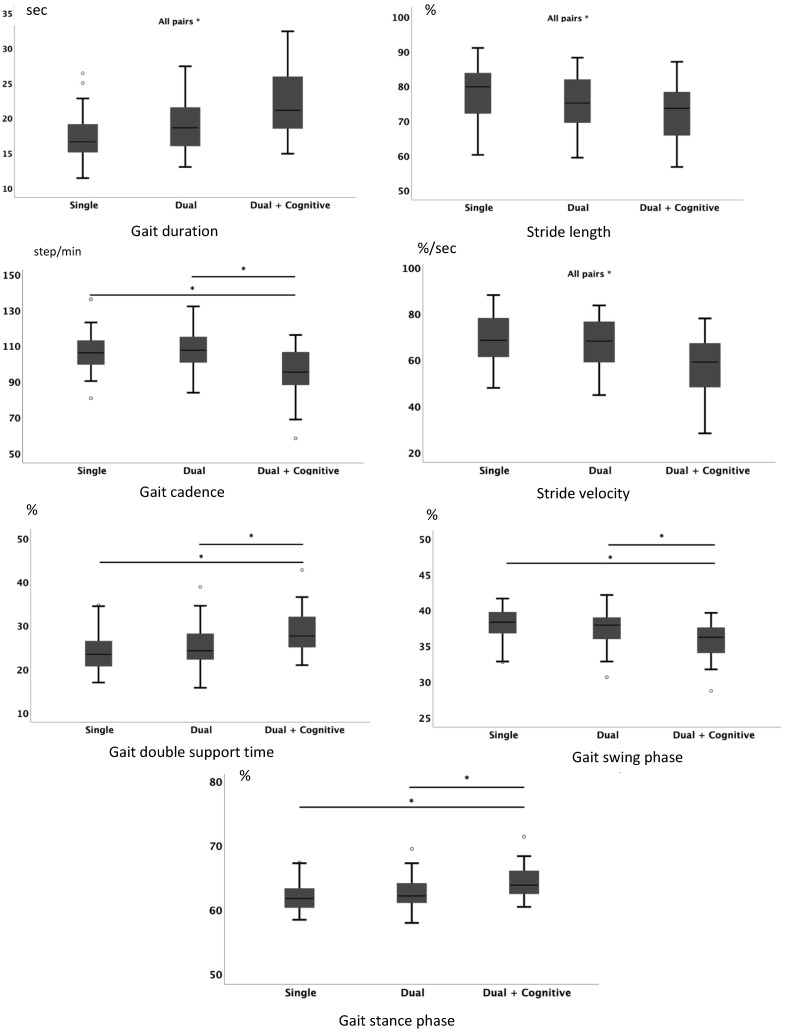
Gait parameters for the three conditions: single task, dual task, and dual and cognitive task during the ITUG test. * Significant differences for pairwise comparisons.

Figure 4 shows significant differences in pairwise comparisons for gait parameters and the respective boxplots.

#### 3.2.3. Turning Phase

Turning duration showed significant differences between all the conditions (*F*_(2,64)_ = 87.413; *p* < 0.001), with an increase in time corresponding to the level of complexity of the task. For all other turning-related assessments, there was a consistent pattern of statistically significant changes when comparing the single motor task to the dual motor task activity but no statistically significant changes when comparing the dual motor task with the dual motor task with cognitive demands conditions.

**Figure 5:**
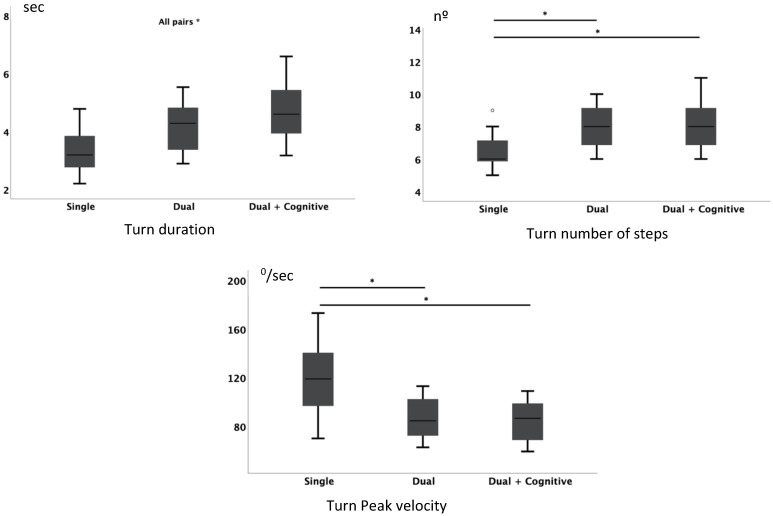
Turning parameters for the three conditions: single task, dual task, and dual and cognitive task during the ITUG test. * Significant differences for pairwise comparisons.

The number of steps revealed significant differences between the different conditions (*χ*^2^ (2) = 42.365; *p* < 0.001; *N* = 32), with the single motor task condition presenting smaller values in relation to the other two dual motor task conditions. No statistically significant differences in the number of steps were identified during the two dual motor task conditions themselves (Figure 5).

Similarly, highly significant differences in peak velocity were identified comparing the single motor task to both other dual motor task conditions (*F*_(1.349, 41834)_ = 72.817; *p* < 0.001). There were no statistically significant differences observed between the two dual motor task conditions.

Figure 5 shows significant differences in pairwise comparisons for turning parameters and the respective boxplots.

#### 3.2.4. Turn-to-Sit Phase

**Figure 6:**
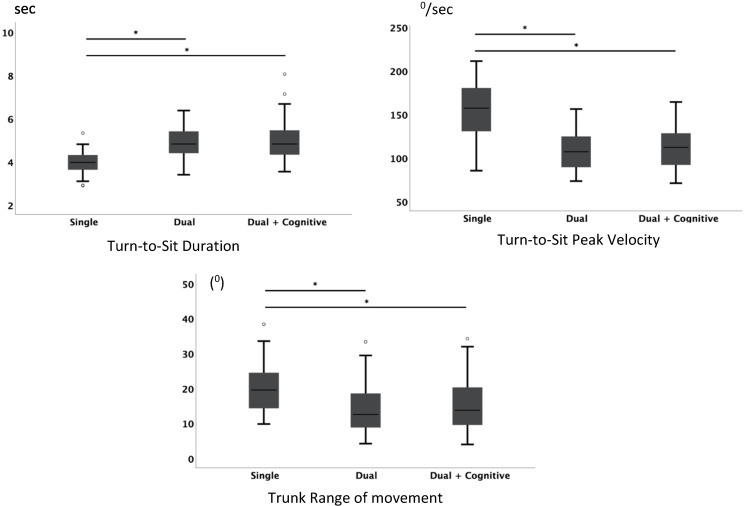
Turn-to-sit parameters for the three conditions: single task, dual task, and dual and cognitive task during the ITUG test. * Significant differences for pairwise comparisons.

Turn-to-sit duration revealed also significant differences only between the single motor task and the two dual motor tasks conditions (*χ*^2^ (2) = 33.515; *p* < 0.001; *N* = 33), with no statistically significant differences between the two dual motor task conditions (Figure 6).

Turn peak velocity revealed significant differences between the single motor task condition and both other dual motor task conditions, (*F*_(2,64)_ = 64.977, *p* < 0.001 and *F*_(1.696, 54.267)_ = 49.739, *p* < 0.001, respectively). There were no significant differences between either of the dual motor task conditions.

Trunk range of movement revealed significant differences between the different conditions (𝐹_(1.696, 54.267)_ = 49.739; *p* < 0.001) with the single motor task condition being responsible for these differences, showing a smaller trunk amplitude related to the other two conditions which did not show significant differences.

Figure 6 shows significant differences in pairwise comparisons for turn-to-sit parameters and the respective boxplots.

## 4. Discussion

The process of ageing is associated with functional decline that affects the speed of the executive function processes, which are the main neurocognitive processes for the proper development of daily life activities [16]. Simple actions including walking while talking require people to divide attention between the two tasks. When cognitive functions deteriorate and processing two actions becomes difficult, older people are placed at risk, and independence is compromised [17]. Healthy adults have the ability to develop dual-task activities; however, as a result of the ageing process and/or stages of neurological disorders, the ability to perform multiple tasks can be affected [1,7]. Consequently, preserving and improving a person’s walking ability during other daily life activities is a crucial aim for extending functionality.

This study revealed that a change in walking performance was observed with the increasing path complexity and under dual-task conditions. Recent studies also showed that performing two tasks or dividing one’s attention between tasks can result in the decrease of walking performance not only in healthy adults [9] but also in people with neurological pathologies [7,18]. 

Consistently, dual motor tasks with cognitive demands resulted in the most instances of statistically significant differences between the two types of dual-task activity identified while monitoring static balance and gait. The demands of a single motor task highlighted statistically significant differences when monitoring more complex movements like sit-to-stand, turning, and turn-to-sit, as well as during tasks involved in changes in movement direction or height control. These types of actions are associated with girdle dissociative control, which, in our sample of healthy older adults, is constrained by a counter-control strategy based on a pelvic and scapular girdle blockage. 

Regarding the different conditions and tasks involved, we may consider that standing and walking are motor actions that can be performed under an automatic control, mediated by subcortical structures, releasing our attention to concurrent tasks needing greater engagement like in cognitive operations. 

Previous studies have also shown how older adults under dual task testing contexts show a reduced gait velocity, cadence, and stride time variability [11,12]. Our study is in line with previous research showing that gait performance change deteriorates, and instability increases when a cognitive load is augmented. These results have clinical impact, since low performance in dual-task activities is associated with an increased risk for dementia [19,20], falls, and functional decline [21,22]. Furthermore, in older adults low gait speed is a significant clinical parameter associated with functional decline, falls, morbidity, and survival [23,24]. Significant improvements in the standard walking speed are related with increased subsistence in community dwelling [24]. 

Turning requires dynamic balance control, which often deteriorates with age. Our results suggest that cognitive processes are highly involved in the increased variability identified during tasks where movement is under an automatic control (like in standing or walking) in our sample composed of healthy older adults. However, during more complex activities such as sit-to-stand, turning and turn-to-sit, where the efficiency and control of the secondary motor task (carrying a tray with a full glass of water) implies a need for additional attention, the primary focus appears to be upon the perceptive information and any cognitive task becomes secondary. Nevertheless, motor actions that require the manipulation and control of sliding objects, or changes in body movement direction or heights, may involve a greater need for attention in order to control and accomplish the intended task. When evaluating the performances of the balance tests, also measuring the accuracy of the given additional task may produce more objective data.

Overall, the study results revealed that there was an adaptation in the motor control of the different tasks, and this should be contemplated in active aging programs of physical activity in order to maintain the effectiveness of the different perceptual-motor processes. The promoting training incorporating neuroplasticity principles, must be considered so that the performance capacity to perform dual tasks can be improved [25]. Using frequent and varied repetitions of different specific exercises is necessary for improving or maintaining performance [26,27].

## 5. Conclusions

The results from this study show that dual-tasking influences balance behavior in healthy older adults. By increasing the task complexity with an additional cognitive task, we change balance control. Healthy older adults are prone to focus on the challenging dual motor task or dual motor task with cognitive demands and sacrifice balance performance to some extent.

Further investigations should test the effects of dual task training protocols in older adults’ balance and walking speed. The application of dual task training appears to be promising to improve the use of these parameters. Furthermore, longitudinal studies that assess the detraining effects are also needed. Therefore, we recommend that this assessment should be carried out in future studies and highlight the need to compare older populations with healthy young and adult subjects.

## Data Availability

The data presented in this study are available on request from the first author.
